# Evaluation of polyanionic cyclodextrins as high affinity binding scaffolds for fentanyl

**DOI:** 10.1038/s41598-023-29662-1

**Published:** 2023-02-15

**Authors:** Brian P. Mayer, Daniel J. Kennedy, Edmond Y. Lau, Carlos A. Valdez

**Affiliations:** 1 Physical and Life Sciences Directorate, Lawrence Livermore National Laboratory, Livermore, CA 94550 USA; 2 Nuclear and Chemical Sciences Division, Lawrence Livermore National Laboratory, 7000 East Avenue, Livermore, CA L-090 94550 USA; 3 Forensic Science Center, Lawrence Livermore National Laboratory, Livermore, CA 94550 USA; 4 Biosciences and Biotechnology Division, Lawrence Livermore National Laboratory, Livermore, CA 94550 USA

**Keywords:** Organic chemistry, Supramolecular chemistry, Chemical synthesis, Theoretical chemistry

## Abstract

Cyclodextrins (CDs) have been previously shown to display modest equilibrium binding affinities (*K*_*a*_ ~ 100–200 M^-1^) for the synthetic opioid analgesic fentanyl. In this work, we describe the synthesis of new CDs possessing extended thioalkylcarboxyl or thioalkylhydroxyl moieties and assess their binding affinity towards fentanyl hydrochloride. The optimal CD studied displays a remarkable affinity for the opioid of *K*_*a*_ = 66,500 M^−1^, the largest value reported for such an inclusion complex to date. One dimensional ^1^H Nuclear Magnetic Resonance (NMR) as well as Rotational Frame Overhauser Spectroscopy (2D-ROESY) experiments supported by molecular dynamics (MD) simulations suggest an unexpected binding behavior, with fentanyl able to bind the CD interior in one of two distinct orientations. Binding energies derived from the MD simulations work correlate strongly with NMR-derived affinities highlighting its utility as a predictive tool for CD candidate optimization. The performance of these host molecules portends their utility as platforms for medical countermeasures for opioid exposure, as biosensors, and in other forensic science applications.

## Introduction

Fentanyl-based synthetic opioids have found numerous applications in the medical field as effective anesthetics and are considered safer than morphine during perioperative procedures^1–3^. Their potent activity stems from their ability to bind various nociceptive receptors in the nervous system, with one of their main targets being the μ-opioid receptor^4,5^. Like morphine and other alkaloids designed to combat pain, fentanyls unfortunately display high risks of dependency and addiction due to their euphoric effect on the central nervous system (CNS). Unfortunately, their illicit use has led to a high rate of overdose-related death in what is largely recognized as a fentanyl epidemic^6–9^.

**Figure 1:**
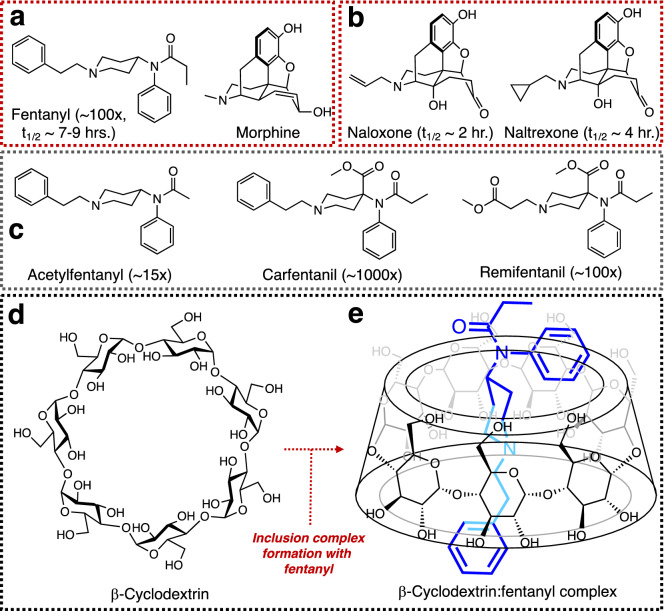
(**a**) Chemical structures of fentanyl and morphine, the potency of the opioid relative to morphine (~ 100x) is provided along with its systemic circulation half-life (t_1/2_); (**b**) structures of two of the most common antidotes used to treat fentanyl poisoning, naloxone and naltrexone along with their systemic circulation half-lives; (**c**) structures of fentanyl analogs encountered in several overdose cases in the US (acetylfentanyl) as well as others used as incapacitating agents (carfentanil and remifentanil), their potencies relative to morphine are provided; (**d**) structure of β-cyclodextrin showing the seven glucose units linked in an α1,4-fashion giving rise to a conical structure (frustrum) open at both ends with a hydrophobic interior and a hydrophilic exterior; (**e**) representation of a hypothetical inclusion complex formed between β-CD and fentanyl.

The flagship compound in this opioid class is fentanyl, which has a potency of ~ 100 times that of morphine (Fig. 1a)^10,11^. Though both fentanyl and morphine display toxic potential if not carefully administered to an individual, fentanyl is often of higher concern due to its much simpler production. Furthermore, given the opioid’s ease of synthesis, the production of novel analogs characterized by increased analgesic profiles is not difficult^12–14^. In addition to their medical uses, some of these opioids such as acetylfentanyl have been linked to fatal overdose cases from recreational use, while carfentanil and remifentanil have been linked to use as chemical warfare agents (Fig. 1b)^15,16^. As fentanyl-based opioids have increased in both potency and use over the last several decades^17^, significant research has focused on the development of efficient analytical methods for their detection^9,16,18^ and on effective antidotes to counteract their physiological effects^19,20^. Antidotes such as naloxone and naltrexone (Fig. 1c) have become the principal methods of overdose treatment, however, they still lack the ability to provide prolonged protection against these synthetic opioids. Such reduced effectiveness is due to the opioid’s long circulation half-life in the blood (t_1/2_ ~ 7–9 h) whereas the circulation half-lives for naloxone and naltrexone are significantly shorter at t_1/2_ ~ 2 h and t_1/2_ ~ 4 h, respectively^19^. To address this issue, research groups have launched programs aimed at developing medical countermeasure (MCMs) candidates that provide longer-term protection against fentanyls, particularly when the affected individual is removed from emergency medical intervention. Some of these efforts have revolved around the notion of employing host molecules that could in principle capture synthetic opioids in their interior and depending on their binding affinity towards it, these could be effective scaffolds to be used as MCMs. One class of host systems that has been studied in detail by the Isaacs group at the University of Maryland for this purpose and also for the reversal of anesthesia has been the cucurbit[n]urils^21,22^. Their work that started with these cyclic-based hosts led to the eventual discovery of a powerful host for methamphetamine^23^ and fentanyl^24^ was curiously acyclic in nature, therefore providing it with enough flexibility to modify its effective trapping of the opioid. Another set of similar hosts but based on a carbohydrate backbone are the cyclodextrins, which unfortunately have been marginally evaluated for these applications^25^.

Cyclodextrins (CDs), cyclic oligosaccharide motifs composed of glucosyl units linked together via α1,4-glycosidic bonds (Fig. 1d), have recently garnered attention as potential medical countermeasure candidates. The cyclic arrangement of these glucose units gives rise to a macromolecular scaffold that resembles a frustrum with a bottom rim larger than its top (Fig. 1e). This arrangement provides CDs with a hydrophilic exterior and a hydrophobic interior able to accommodate organic molecules, a key physical property of these compounds^26,27^. This ability to serve as a molecular host has made CDs versatile platforms for use in industry^28^, medicine^29^, and materials science^30^. Naturally, the larger the number of glucose units, the larger the frustrum’s cavity is and thus its ability to host larger guests in its interior. A crucial and useful experimental handle researchers have is the host:guest complexes, also called inclusion complexes, can be studied by various spectroscopic methods that include UV-Vis^31^, infra-red^32^, and nuclear magnetic resonance spectroscopies^33^ as well as spectrometric methods like liquid chromatography mass spectrometry (LC–MS)^34^. Our previous work has relied principally on NMR to study complexes between native CDs and fentanyl and other toxins (Fig. 1e)^25,35^. Making use of extensive NMR titration studies in conjunction with two-dimensional Rotational Frame Overhauser Spectroscopy (2D-ROESY), one can determine CD:fentanyl binding affinities as well as the structure of the complex. Even though it was previously demonstrated inclusion complexes form between native CDs and fentanyl, the binding affinities were low and not significantly strong to make a case for their use as a potential fentanyl binder. In this report, we present the synthesis of more complex CD scaffolds observed to form strong inclusion complexes with fentanyl. Our efforts have leveraged NMR titrations to determine the binding strength between the current lead candidate, called “subetadex” (SBX), and fentanyl. In addition, important insight into the structure of the SBX:fentanyl complex was accomplished using 2D-ROESY NMR experiments. These experimental data were found to be in good agreement with computational modeling calculations that were also used to explore additional CD scaffold candidates with enhanced fentanyl affinities.

## Results and discussion

This work focuses on the exploration of more complex CD scaffolds encouraged by our initial studies on the capture of fentanyl with native and simply substituted CDs^25^ as well as the knowledge of other CD scaffolds that have successfully found major biomedical applications for their ability to bind and effectively neutralize specific targets. One such CD is Sugammadex (SGX), which is a FDA-approved drug for the treatment of patients who experience prolonged post-operative anesthetic effects of rocuronium bromide (RocBr)^36–40^. SGX was discovered in 2001 at Organon Laboratories^36^ and displayed a strong affinity for RocBr while minimizing potentially toxic side effects. After years of evaluation, SGX was approved by the FDA in 2015 and has now become a common treatment to alleviate the anesthetic symptoms associated with the use of RocBr in the surgical setting due to its non-toxic nature and the strong complex association constant for the anesthetic (*K*_*a*_ ~ 750,000 M^-1^) determined by nuclear magnetic resonance (NMR) titration experiments^41^. Like SGX, the present work sought to identify a CD candidate with a non-toxic profile while displaying a high affinity for fentanyl and related synthetic opioids.

**Figure 2:**
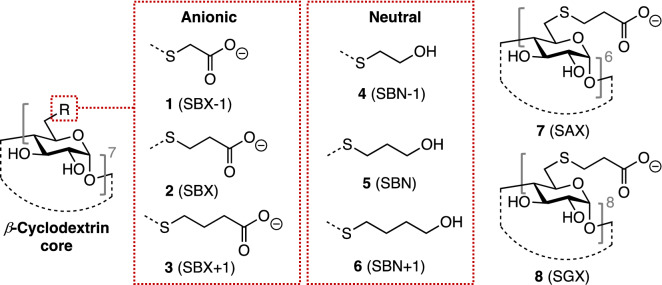
Basic structural scheme of a βCD with its upper rim, C6-hydroxyl moiety replaced with a generalized R group. There are two types of moieties investigated in this work, the anionic ones comprising CDs featuring the 2-mercaptoacetyl (SBX-1, **1**), 3-mercaptopropionoyl (SBX, **2**) and 4-mercaptobutanoyl (SBX + 1, **3**) groups. The second type of moieties involve ones bearing neutral moieties in the upper rim featuring the 2-mercaptoethanol (SBN-1, **4**), 3-mercaptopropanol (SBN, **5**), and 4-mercaptobutanol (SBN + 1, **6**). In addition to these βCD-based targets, SAX (Sualphadex, **7**) and SGX (Sugammadex, **8**) were also synthesized and evaluated separately, however, both did not show any significant binding to fentanyl.

Structurally, SGX is based on a γ-CD core having its upper rim chemically modified with 3-mercaptopropanoic acid chains. These additional moieties serve to elongate the native CD hydrophobic interior. Based on our previous studies^25^, the γ-CD cavity was found to be too large to accommodate fentanyl in its interior, and MD calculations showed the opioid spent the majority of the simulation time unbound. For this reason, initial screening efforts were focused on β-cyclodextrin-(β-CD)-based scaffolds, as their interior better accommodated the opioid. Our previous NMR data and MD simulations both showed fentanyls bind native β-CD at the fentanyl amide phenyl ring, though observed binding affinities were quite low (*K*_*a*_ < 250 M^-1^). The structures of these complexes determined from these data were found to leave the majority of the fentanyl molecule outside of the CD, with its phenethyl group largely solvated by water. These enthalpically unfavorable interactions in part explain in the observed weak complexes*.* To improve complexation, CDs were envisioned that enclose a far greater fraction of the opioid thereby providing more favorable interactions, principally van der Waals and Coulombic^34^. The library screened in this current work is based on the β-CD host with slight variations on a common modification, namely the replacement of all 6-position hydroxyl groups with straight-chain thioalkylcarboxylates or thioalkylalcohols (Fig. 2).

Equilibrium binding affinities between the CD panel and fentanyl·HCl were determined using ^1^H NMR titration experiments. In order to derive the most accurate binding constant, experiments were designed such that the CD concentration [*H*]_*T*_ (in units of M) satisfied the inequality [*H*]_*T*_* K* < 1 whenever possible. Initial studies required best-guess estimates of *K* since they were a priori unknown. The ^1^H NMR chemical shifts of reporter protons on CD (H3 and H5) were monitored while the fentanyl titrant solution was added incrementally. Representative data from these titrations are found in Fig. 2 of Supporting Information. Non-linear least squares fitting was performed using mathematical expressions appropriate for the binding data obtained. This fitting was performed on all reporter proton titration curves simultaneously yielding a global binding affinity for each CD:fentanyl pair^25^. Results from this fitting procedure are given in the first data column of Table 1.

**Table 1:** NMR-derived binding affinities and energies from molecular dynamics simulations for CD:fentanyl complexes. SAX and SGX did not demonstrate any significant binding to fentanyl.

	NMR simple 1:1	NMR two-state 1:1		MD Results	
CD	*K* / mM^− 1^	*K*_1_ / mM^− 1^	*K*_2_ / mM^− 1^	$\langle U+W\rangle$_up_ / kcal mol^− 1^	$\langle U+W\rangle$_down_ / kcal mol^− 1^
SBX-1	21.7 ± 7.0	–^b^	–^b^	− 27.7 ± 0.5	− 30.8 ± 1.4
SBX	30.1 ± 6.2	44 ± 22	2.1 ± 1.1	− 31.7 ± 0.6	− 30.8 ± 1.2
SBX + 1	–^a^	67 ± 23	2.5 ± 0.5	− 38.6 ± 2.8	− 32.8 ± 0.6
SBN-1	2.7 ± 0.2	–^b^	–^b^	− 33.6 ± 1.4^c^	–
SBN	1.6 ± 0.1	–^b^	–^b^	− 35.5 ± 1.2^c^	–
SBN + 1	0.2 ± 0.1	–^b^	–^b^	− 37.6 ± 1.8^c^	–

^a^1:1 model does not hold.

^b^More complex model not necessary to fit titration data.

^c^Only “up” orientation of fentanyl simulated.

In most cases, the NMR data could be fit to a model that describes the binding as a simple 1:1 system. NMR Job plot data, however, which is used to justify the use of such a model showed distinct deviation from a 1:1 binding model for both SBX and SBX + 1, with maxima slightly off from the expected fraction, r = 0.5 (see Fig. 1 in Supporting Information). These data urged the consideration of a two-state 1:1 model which was able to describe both the observed Job plot behavior and that from the titrations themselves. Additionally, the titration data showed distinctly non-monotonic behavior for multiple reporter protons (see Fig. 2 in the Supporting Information).

**Figure 3:**
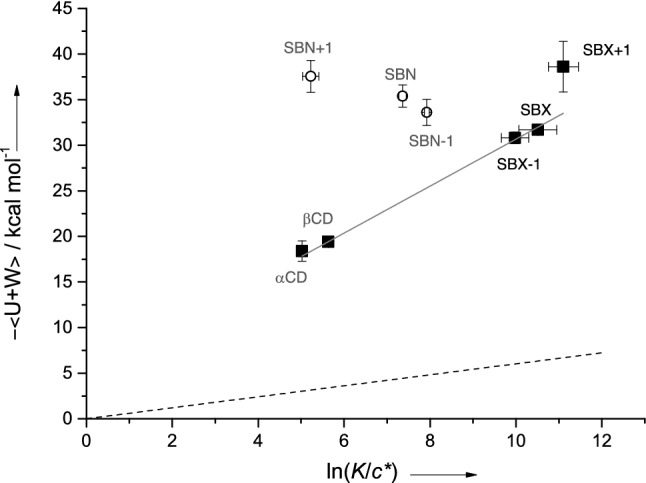
NMR binding affinities (simple 1:1 model) versus enthalpic energies determined from simulation for the dominant conformer of fentanyl bound to hosts **1**–**6** and to α- and β-cyclodextrin^31^. Note the affinity reported here for SBX + 1 is the dominant conformation from the more complex two-state 1:1 model (67 mM^-1^) Neutral CD scaffolds given as circles; all others as filled squares. The solid line is a linear fit to filled data points considering measurement uncertainties. The dashed line has slope RT (0.602 kcal mol^-1^ at 303 K).

Figure 3 compares NMR-determined binding affinities for 1:1 CD:fentanyl complexes to MD simulation binding energies (values given in right-most columns of Table 1). Data for α-CD and β-CD from our previous work are included for completeness. For the anionic SBX-1, SBX and SBX + 1 (**2**–**4**), NMR results show strong correlation with behavior predicted by the MD simulations. That is, binding affinity increases with binding energy likely due to enhanced van der Waals interactions afforded by the hydrophobic nature of the elongated backbone of the anionic thioalkylcarboxylates. This assertion is supported by additional computational work showing an plateau in affinity for CDs displaying further-extended anionic arms (*e.g.,* SBX + 2 and SBX + 3 (not synthesized)). This observation indicates there is little advantage to thiocarboxylate arms longer than four carbons (*i.e.*, SBX + 1), which are sufficient to encase completely the fentanyl molecule (*i.e.,* optimal size complementarity). We refrain, however, from overinterpreting any direct NMR/MD comparisons, as MD-derived quantities neglect the effect of conformational entropy in the overall thermodynamics. In other words, a free energy is not strictly calculated. Instead, an average energy is calculated, referred to as < *U* + *W* > as previously described^41^.

For the neutral CDs (*i.e*., SBN-1, SBN and SBN + 1) initial MD simulations predicted an enhanced binding affinity with increasing arm length in similar fashion to their anionic counterparts. NMR data, however, show a dramatic decrease in binding affinity, one that actually worsens with increasing arm length (see Table 1, Fig. 1). NMR structural data from ROESY experiments show clear correlations between the CD’s interior H3 and H5 protons and those along its thioalkylhydroxyl arms (see Supporting Information). Additional MD simulations of these neutral CDs alone in solution supported this behavior. Ultimately, the hydrophobic driving force that exists for the hydrophobic thioalkylhydroxyl arms to orient themselves inside the CD cavity seems to preclude fentanyl from binding via a competitive, self-binding effect with longer arms binding better within the cavity. We then infer the thiocarboxylate arms of the charged CDs not only provide the necessary aqueous solubility of the CD but, arguably more importantly, likely hold the anionic SBX open wide enough for fentanyl to bind via electrostatic repulsion. Note MD suggests there is little interaction between the carboxylate terminuses and the fentanyl molecule, as the charged groups orient into their aqueous surrounding.

**Figure 4:**
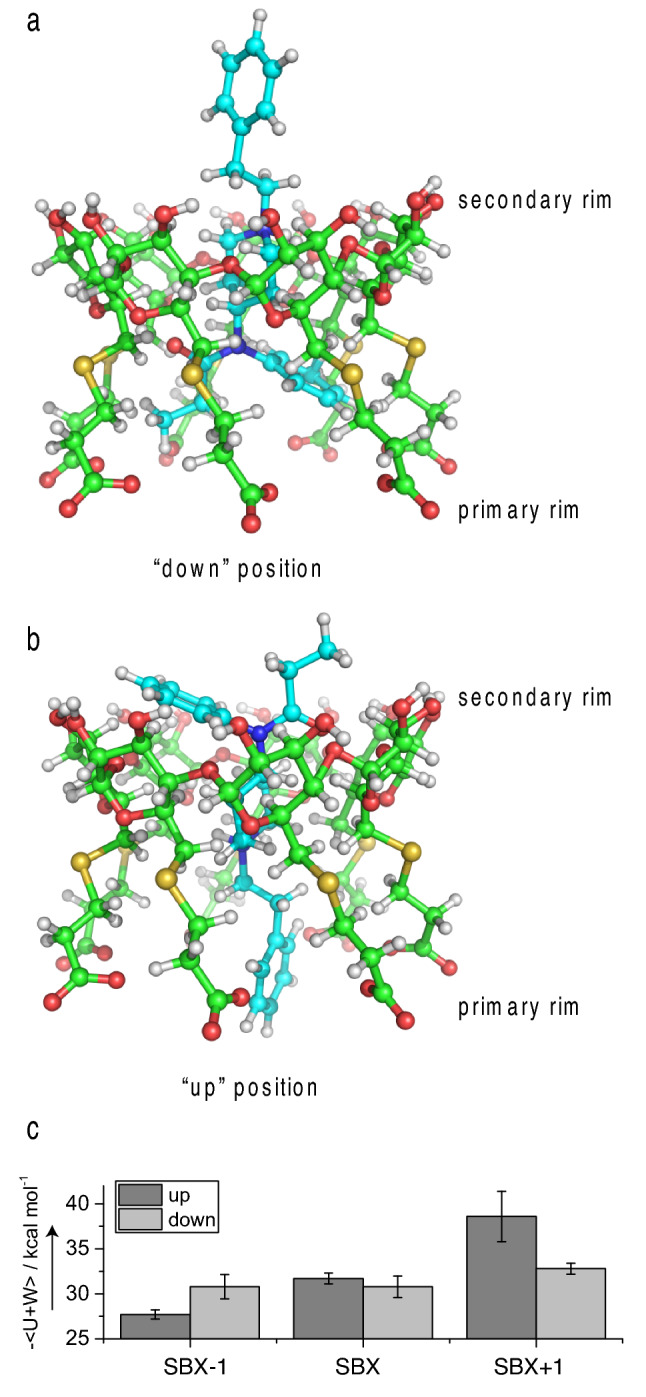
MD results for the host:guest complex with two orientations: (**a**) fentanyl (carbon atoms in cyan) aligned “down” with amide half near primary rim of SBX (carbon atoms in green), (**b**) fentanyl in alternate, “up” position within SBX, c) binding energies for all three SBX complexes.

The results presented thus far represent a simplified view of structural details of the SBX:fentanyl complexes. As mentioned earlier, NMR data in the form of Job plots (see Fig. 1 in Supporting Information), which are used to determine host:guest stoichiometries^42–44^, revealed more complex binding behavior initially thought to originate from a 1:2 or 2:1 host:guest complex. MD simulations, however, showed CD could not accommodate a second fentanyl molecule. Additional simulations, however, showed the charged CDs could accommodate fentanyl in two distinct conformations (Fig. 4). In the case of SBX-1, the orientation with the amide half of fentanyl pointed “down” toward the anionic chain ends is slightly energetically favored over fentanyl in the opposite direction (see Fig. 4a). For SBX + 1, the reversed configuration is favored with the amide half pointed “up” toward the unmodified secondary rim of CD (see Fig. 4b). Both of these conformations were observed to be favorable, with relatively small differences in energies between them (0.8–5.8 kcal mol^-1^ depending on host molecule, Fig. 4c). For SBX, the two conformations are equally favorable.

**Figure 5:**
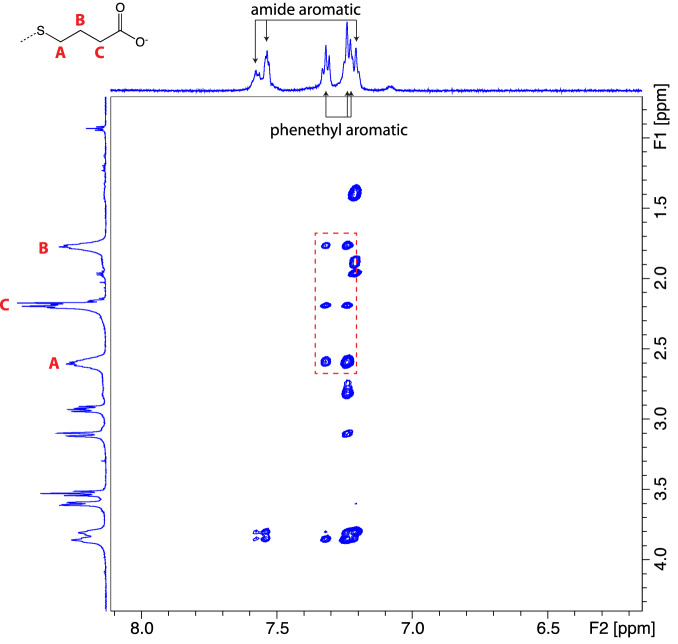
Partial ROESY spectrum of SBX + 1:fentanyl HCl complex. Correlations between fentanyl aromatic protons from the phenethyl ring and SBX + 1 methylenes are highlighted by the dashed box. Full spectrum is given in Fig. 3 of Supporting Information.

MD findings are consistent with rotating-frame nuclear Overhauser effect spectroscopy (ROESY) NMR experiments. Figure 5 gives the two-dimensional ROESY spectrum of a SBX + 1:fentanyl mixture (see also Fig. 3 in Supporting Information). Of particular importance is the complete absence of correlations between methylene protons on the anionic CD arm and aromatic protons on the amide nitrogen-bound phenyl ring. Rather, only correlations between these methylenes and the phenethyl aromatic protons of fentanyl were found. This finding indicates the majority of fentanyl binds in the “up” orientation, consistent with MD results. ROESY data for a SBX-1:fentanyl mixture shows a similar agreement to MD data, namely, amide phenyl protons correlate to interior CD protons near its unmodified, secondary rim. These peaks provide clear evidence for a preference of the complex to adopt the “down” orientation. For all three charged CDs, Job plot data (see select data in Fig. 1 of Supporting Information) display evidence of a two-state binding system regardless of a lack of clear correlations in the ROESY spectra. Job plots are much more sensitive at detecting these types of phenomena, as they cover a substantially wider relative concentration range (*i.e*. [*H*]_0_/[*G*]_0_ ratio) than titration experiments.

Evidence of an equilibrium coexistence of the two conformations implies the 1:1 binding model invoked previous is not strictly appropriate for the extraction of equilibrium binding affinities. We therefore developed a fitting model that explicitly considers both orientations on the chemical shift titration data. Details of this “self-competitive” model are given in Supporting Information. The results of fitting to such a model yielded two equilibrium binding constants. For SBX:fentanyl, the larger of the two *K*_*a*_ values was observed to be statistically similar to that derived from the 1:1 model. Secondary binding constants, indicative of the weaker complex, are also extracted, but it was possible with titration data alone to unambiguously assign affinities to particular conformations due to its associated relative population being so low. The apparent agreement of NMR ROESY data with the MD results, however, seems to suggest the dominant conformations established from NMR data are those with the highest computed energies (*i.e*., most enthalpically favorable) as given in Fig. 3. However, the fitting parameters from this more physically relevant fit are much less robust than those obtained from the 1:1 model due to the number of and high correlation among fitting parameters. For this reason, we plotted binding constants obtained for the more robust 1:1 model in Fig. 1 (with SBX + 1 being the exception since the data could not be fit reasonably well with the simple model).

## Experimental section

### Materials

Reagents and solvents were purchased and used as received. Methyl 3-mercaptopropionate, cesium carbonate, and 2-mercaptoethan-1-ol were purchased from Alfa Aesar (Ward Hill, MA). Methyl thioglycolate was purchased from Pfaltz and Bauer, Inc. (Waterbury, CT). Methyl 4-sulfanylbutanoate was purchased from Enamine Ltd. (Kyiv, Ukraine). *N*-methyl-2-pyrrolidone was purchased from Applied Biosystems, Thermo Fisher (Grand Island, NY). 4-Mercaptobutan-1-ol and 3-mercaptopropan-1-ol were synthesized as previously described^45,46^ purified by flash column chromatography in silica gel cartridges with a Biotage Isolera purification system, and their purity assessed by ^1^H NMR and GC-MS^47–49^. Deuterated water (D_2_O) was purchased from Cambridge Isotope Laboratories, Inc. (Tewksbury, MA). Hexakis-, heptakis- and octakis-C6-brominated cyclodextrins were purchased from Arachem/Cyclodextrin Shop (Tilberg, Netherlands). Thiol-mediated displacement reactions on the C6-per-brominated cyclodextrins followed by methyl ester hydrolysis (where applicable) were accomplished with a modified version of the protocol originally published by Adam *et al*^36^. For the synthesis of mercaptoalkylcarboxylic acid methyl ester intermediates, thin layer chromatography (TLC) was used to monitor their production using Merck kieselgel 60-F254 sheets and detection accomplished with UV light (λ = 254 nm) in conjunction with development of color with Ce(SO_4_)_2_/(NH_4_)_6_Mo_7_O_24_·4H_2_O/H_2_SO_4_/H_2_O (CAM)^50–53^ and iodine vapor^54–56^. Centrifugation was performed in a Eppendorf centrifuge model 5810R at 3220 rpm for 5 min, using Falcon tubes (50 mL) purchased from VWR (Aurora, CO.). Solvents were removed using an IKA RV8 model rotary evaporator coupled to a KNF-Lab vacuum filtration pump and a VWR RS-232 cooling/circulating system (9 °C water/ethylene glycol 1:1).

### Nuclear magnetic resonance and other analytical characterization

^1^H NMR (600 MHz), ^13^C NMR (150 MHz) and ^13^C-DEPT NMR (150 MHz) spectra were recorded in D_2_O unless otherwise specified. Spectra were obtained using a Bruker Avance III 600 MHz instrument equipped with a Bruker TCI 5 mm cryoprobe (Bruker Biospin, Billerica, MA) at 30.0 ± 0.1 °C. NMR data for the synthesized compounds detailed below are reported as follows: chemical shift (δ) (parts per million, ppm); multiplicity: s (singlet), d (doublet), t (triplet), dd (doublet of doublets), td (triplet of doublets), m (multiplet), app (apparent) and br (broad); coupling constants (*J*) are given in Hertz (Hz). For the experimental data used in characterization of host:guest complexes, One-dimensional ^1^H NMR data were collected with water suppression by excitation sculpting with gradients^57^. For each 1-D experiment, 16 to 128 transients (depending on analyte concentration) were collected using 65,536 data points, a 4.0 s acquisition time, and a 1.0 s relaxation delay. Prior to the Fourier transformation the free induction decays (FIDs) were apodized with an exponential decay equivalent to 0.25 Hz line broadening. Two-dimensional ROESY spectra were acquired using 16,384 data points with 1024 increments, 8—32 scans for each increment, and a continuous wave spin lock with a 200 ms mixing time and a frequency of 3.57 kHz. Further details can be found in our previous publication^25^. Exact masses of the synthesized compounds below were obtained on a Bruker micrOTOF-Q III (Bruker Daltonics, Billerica, MA) equipped with an electrospray ionization (ESI) source. Time-of-flight mass spectroscopic detection was performed in negative ion mode. Mass calibration was performed in the final minutes of each run using Agilent’s ESI-L Low Concentration Tuning Mix (G1969-85,000). An Agilent 1260 Liquid Chromatograph (LC) equipped with an Atlantis T3 Reverse Phase Column (C18, 150 mm × 2.1 mm, 3 μm particle size, Waters, Milford, MA) was used for the separation of all compounds. The LC mobile phase consisted of water with 0.1% formic acid (A) and acetonitrile with 0.1% formic acid (B). The gradient profile started with 95% A for 2 min, ramped to 5% A at 18 min, held for 13.5 min, ramped quickly back to 95% over 0.5 min, and held for 10 min for column regeneration. This method was used for all samples except those for the vinyl tile surface study, for which the final 5% A was held for 18.5 min. Ten microliters of the liquid sample were introduced via an autosampler (Agilent B1329B) to the injection port.

### NMR data processing and binding constant determinations

Equilibrium binding constants between the CD family and fentanyl HCl were determined using ^1^H NMR titration experiments. In order to derive the most accurate binding constant, the cyclodextrin concentration [*H*]_*T*_ (in units of M) satisfied the inequality [*H*]_*T*_*K* < 1 whenever possible. Initial studies required best-guess estimates of *K* since they were a priori unknown. Ultimately, this condition was satisfied for all compounds save for SBX + 1, where the required concentration yielded an unacceptable signal-to-noise ratio. Use of this non-ideal concentration leads to a larger uncertainty in the derived binding constant (vide infra*)*. Due to the limited aqueous solubilities of the neutral compounds **5**–**7** (< 1 mg·mL^-1^ in all cases), all titrations were conducted at [*H*]_*T*_ < 0.1 mM. Each of the modified CD was dissolved into deuterated water (D_2_O) to a total volume of 500 μL with 1 μL of acetonitrile added as a chemical shift reference point. Fentanyl HCl was then titrated into the solution. The total (bound to cyclodextrin + free in solution) fentanyl HCl concentration [*G*]_*T*_ (in units of M) was varied to cover the range of concentrations between $\frac{\left(0.2{\left[H\right]}_{T}+0.25\right)}{K}$ and $\frac{\left(0.8{\left[H\right]}_{T}+4\right)}{K}$ in order to allow accurate extraction of binding constants. At each titration point, the proton chemical shifts (referenced to acetonitrile at 2.014 ppm) of the H1, H3, H5, and H6 protons on the CD were recorded; subtraction of the chemical shift obtained from the first data point, consisting of the cyclodextrin alone, yielded a difference between bound and free chemical shift $\Delta {\delta }_{i}$ for each of reporter proton *i*. The resultant curves of $\Delta {\delta }_{i}$ versus [*G*]_*T*_ were then initially fit (Mathematica 10, Wolfram) using an non-linear least squares algorithm discussed previously (see additional mathematical details in Supporting Information)^31^. As in our previous work, the binding constant *K* and a chemical shift sensitivity factor $\Delta {\updelta }_{c,i}$ for each reporter proton are the fitting parameters. Restricting *K* to a single value for each NMR titration curve yields a global binding constant incorporating data from all reporter protons^58^. All experiments were conducted in triplicate. Reported values of *K* for each compound are the average and standard deviation across the three experiments.

### Molecular dynamics simulations

Molecular dynamics (MD) were performed with AMBER^59^ (version 12) employing the recent charges and parameters applied for cyclodextrin scaffolds^60,61^ and the GAFF force field for the various fentanyls^62^ in their protonated states^63^. Fentanyl charges were generated by AM1-BCC calculations^55^ in the program ANTECHAMBER. The program CHIMERA was employed for modeling the CD:fentanyl complexation processes^64^. The CD and CD:fentanyl complexes were solvated in a box of TIP3P water sufficient in size to have at least 15 Å of water between the solute and the solvent interface (~ 51 × 51 × 51 Å^3^ initial box size)^65^. To neutralize the systems, sufficient sodium (Na^+^) ions (typically one) were added to the system. The systems consisted of about 12,500 atoms (~ 4,100 water molecules). Each system was energy minimized using 250 steps of steepest descent and 1500 steps of conjugate gradient. Constant temperature and pressure dynamics (NPT) were performed on these minimized systems^66,67^. Periodic boundary conditions were used, and electrostatic interactions treated by particle mesh Ewald methods using a 9 Å cut-off in direct space with a 1 Å grid^68^. Bonds containing hydrogen were constrained using SHAKE and a time step of 2 fs was used for each simulation^69^. The systems were initially coupled to a heat bath at 100 K for the first 100 ps, then increased to 200 K for the next 100 ps, and finally raised to 300 K for the remainder of the simulation. Each simulation was performed for a total of 10 ns or 30 ns depending on the CD guest. Initial simulations involved only single trajectories, but it was universally observed that the βCD structure did not change significantly during the simulation, and that the root mean squared deviation of the non-hydrogen atoms relative to the average structure quickly plateaued by 1 ns. The first 2.5 ns of the 300 K dynamics were used for equilibration. To obtain more comprehensive data and statistical information, ten replicate simulations (10 ns each) were performed for complexes between all four charged fentanyls and βCD where the guest molecule was parallel to the wider CD rim. These replicate simulations were characterized by changing the seed value for generating the initial velocities for the system.

The free energy of binding between the cyclodextrins and fentanyls were estimated using the Molecular Mechanics-Generalized Born Surface Area (MM-GBSA) method from snapshots of the solvated trajectories^70–73^. MM-GBSA energy calculations were performed on replicate simulations and then averaged to obtain the average binding energy for a particular fentanyl:CD system. The binding free energy was estimated by the equation:

$$ \Delta G_{binding} = G_{complex} {-} \, \left( {G_{CD} + G_{fentanyl} } \right) $$

were each term, *G* is estimated as the sum of gas-phase molecular mechanics energy *E*_*gas*_ and the solvation energy *G*_*sol*_:

$$ G_{total} = \, E_{gas} + G_{sol} $$

The contribution of entropy was neglected in these free energy calculations. The solvation free energy (*G*_*sol*_) is the sum of the polar and nonpolar solvation energies of the molecules determined by solving the Generalized Born (GB) equation. The binding free energies for the complexes were calculated using the MMPBSA.py script in AMBER12 on snapshots from each 7.5 ns trajectory. The modified Onufriev-Bashford-Case-I GB (ib = 2) model was used for the calculation with a fentanyl salt concentration of 0.24 mM^74^. The surface tension used to calculate the nonpolar contribution to the free energy of solvation was 0.0072 kcal mol^-1^ Å^-2^.

## Conclusions

In this work, we have studied the inclusion complexes between a number of chemically modified CDs with the synthetic opioid fentanyl. The evaluated panel of CDs exhibited either anionic thioalkylcarboxylic acid side chains or neutral thioalkyl alcohols side chains on their top rim. The charged CDs were found to have extended hydrophobic interiors kept open by mutual electrostatic side chain repulsion, thus providing a sufficient binding pocket for the opioid. MD simulations of the SBX:fentanyl inclusion complexes were found to be consistent with binding affinity trends extracted from NMR experiments. That is, binding strength increases as the length of the modified arm increases for the three charged CDs studied. For CDs with neutral thioalkyl side chains, binding affinities were observed to decrease with increasing arm length, likely a result of competitive binding of the more hydrophobic neutral side chains also seeking the hydrophobic SBN interiors. MD simulations also supported this competitive binding phenomenon revealing that longer arms tended to interact more strongly with the hydrophobic interior. For the panel of promising charged SBXs, the maximum fentanyl binding affinity was found to be *K*_*a*_ = 66,500 M^-1^ for SBX + 1. To our knowledge this is the largest equilibrium binding affinity reported for CD:fentanyl complexes. We believe that these CD scaffolds could be developed into promising, novel medical countermeasures candidates against these potential deadly opioids. Another important avenue for future investigation but is not covered presently: the potential power of simultaneously fitting Job plot and titration data to extract more confident values of highly correlated fitting parameters. Our group is also focusing on exploiting these compounds for environmental scavenging of fentanyls as well as for detection of these opioids in complex biological forensic samples.

## Data and materials availability

All data generated or analyzed during this study are included in this published article and its supplementary information files.

## Supplementary Information

Supplementary Information.
