# Supplementary material for: Evaluation of polyanionic cyclodextrins as high affinity binding scaffolds for fentanyl
Source: Sci Rep. 2023 Feb 15;13:2680. doi: 10.1038/s41598-023-29662-1 (PMC9932099; doi:10.1038/s41598-023-29662-1)
Supplement: Supplementary file 1 — Supplementary Information. [file 41598_2023_29662_MOESM1_ESM.docx]

**-Supporting Information-**

**Evaluation of Polyanionic Cyclodextrins as High Affinity Binding Scaffolds for Fentanyl**

Brian P. Mayer^1-3^, Daniel J. Kennedy^1-3^, Edmond Y. Lau^1,2,4^ & Carlos A. Valdez^1-3,^*

^1^Physical and Life Sciences Directorate, Lawrence Livermore National Laboratory, Livermore, CA, 94550, USA.

^2^Nuclear and Chemical Sciences Division, Lawrence Livermore National Laboratory, Livermore, CA, 94550, USA.

^3^Forensic Science Center, Lawrence Livermore National Laboratory, Livermore, CA, 94550, USA.

^4^Biosciences and Biotechnology Division, Lawrence Livermore National Laboratory, Livermore, CA, 94550, USA.

**Table of Contents**

Page

Description of Compound Nomenclature and Synthetic Protocols 3

Synthesis of SBX-1 4

Synthesis of SBX 5

Synthesis of SBX+1 6

Synthesis of SBN-1 7

Synthesis of SBN 8

Synthesis of SBN+1 9

Synthesis of SAX 10

Synthesis of SGX 11

Calculations for Binding Models for Inclusion Complex 13

Job plots 16

NMR 2D-ROESY data 17

**Description of Compound Nomenclature and Synthetic Protocols.** Before describing the synthetic routes leading to the various cyclodextrins presented in this work in detail, we would like to provide a brief note on the notation that we use to simplify the naming of each compound. Our starting point is Sugammadex^®^ (**8**), the scaffold featuring a propionic thioalkylcarboxylic acid side chain off each one of the C6 positions of the *γ*-cyclodextrin core, for which we have chosen the short notation SGX. Likewise, for Sualphadex (**7**) and Subetadex (**2**) our notations will be SAX and SBX respectively. With regards to the neutral version of SBX (no carboxylates, only the terminal alcohols but retaining the same number of carbon atoms), we have chosen to denote this as SBN (**5**) with the N standing for Neutral. In addition, when referring to the analogs that possess one carbon less than the 3 featured in SBX, we use the notation SBX-1 (**1**) for the carboxylate form and SBN-1 (**4**) for the neutral counterpart (alcohol). Lastly, analogs featuring one carbon more than the 3 featured in SBX are denoted as SBX+1 (**3**) for the carboxylate form and SBN+1 (**6**) for the neutral counterpart (alcohol).

Heptakis(6-*S*-(carboxymethyl)-6-thio)cyclomaltoheptaose heptapotassium salt (SBX-1, **1**)

Heptakis-6-bromo-6-deoxy-*β*-cyclodextrin (1.2 g, 0.76 mmol) was made into a suspension in *N*-methyl-2-pyrrolidone (NMP, 20 mL) in a 100 mL round bottomed flask equipped with a stir bar. To this solution, cesium carbonate (2.48 g, 7.6 mmol, 10 equiv. to cyclodextrin) was added in small portions followed by the addition via syringe of methyl thioglycolate (0.68 mL, 0.81 g, 7.6 mmol, 10 equiv. to cyclodextrin). The resulting mixture was heated to 55 ^o^C overnight with vigorous stirring. The following day, the suspension was cooled to ambient temperature and the mixture added dropwise to a vigorously stirring 500 mL Erlenmeyer flask filled with 300 mL of acetone to induce precipitation of the modified cyclodextrin. Stirring of the suspension was done at ambient temperature for 10 minutes and the white precipitate was collected first by centrifugation. The white solid was then washed with deionized water (2 x 50 mL) and centrifuged again. The collected precipitate was taken up in acetone (50 mL) and vacuum filtered through a fritted disc filter (medium porosity) and dried under vacuum for 30 minutes to furnish the methyl ester *β*-cyclodextrin intermediate as a pure, off-white solid (1.1 g, 84%). The methyl ester β-cyclodextrin intermediate (1.1 g, 0.63 mmol) was treated with 1 M KOH/H_2_O (4.73 mL, 4.73 mmol, 7.5 equiv. to cyclodextrin) in a 20 mL scintillation vial equipped with a stir bar. The initial suspension became a full solution (light tan in color) after 10 minutes of stirring at ambient temperature. The mixture was vigorously stirred overnight. The light tan solution was added dropwise to a stirring acetone bath (300 mL) in a 500 mL Erlenmeyer flask. White flakes precipitated out upon the addition of the mixture and these were collected by centrifugation. Additionally, the white solid was re-suspended, washed with MeOH (2 x 50 mL) and collected by centrifugation. Again, it was found that the additional methanol wash was found to efficiently remove any remaining KOH in the mixture. Lastly, the solid was vacuum filtered, washed with MeOH (2 x 20 mL) and dried under vacuum for 2 h. The procedure yielded pure heptakis(6-*S*-(carboxymethyl)-6-thio)cyclomaltoheptaose heptapotassium salt (SBX-1, **1**) (1.04 g, 86%). ^1^H NMR (D_2_O, 600 MHz) δ 5.09 (s, 6H), 4.05 (t, *J* = 7.2, 6H), 3.92 (t, *J* = 9.6, 6H), 3.68-3.62 (m, 12H), 3.43-3.36 (m, 12H), 3.16 (d, *J* = 14.4, 6H), 2.97 (app dd, *J* = 14.4, 6.6, 6H); ^13^C NMR (D_2_O, 150 MHz) δ 177.6, 101.8, 83.6, 73.0, 72.4, 71.5, 38.6, 33.7; LC-MS(TOF): [M-2H]^2-^, *m/z =* 825.1158 (825.1157 calc.).

Heptakis(6-*S*-(2-carboxyethyl)-6-thio)cyclomaltoheptaose heptapotassium salt (SBX, **2**)

Heptakis-6-bromo-6-deoxy-*β*-cyclodextrin (1.5 g, 0.95 mmol) was made into a suspension in *N*-methyl-2-pyrrolidone (NMP, 20 mL) in a 100 mL round bottomed flask equipped with a stir bar. Upon gentle heating the cyclodextrin fully dissolved forming a light tan-colored solution. To this solution, cesium carbonate (3.1 g, 9.5 mmol, 10 equiv. to cyclodextrin) was added in small portions followed by the addition via syringe of methyl 3-mercaptopropionate (1.04 mL, 1.14 g, 9.5 mmol, 10 equiv. to cyclodextrin). The resulting mixture was heated to 55 ^o^C overnight with vigorous stirring. The following day, the suspension was cooled to ambient temperature and the mixture added dropwise to a vigorously stirring 500 mL Erlenmeyer flask filled with 300 mL of acetone to induce precipitation of the modified cyclodextrin. Stirring of the suspension was done at ambient temperature for 10 minutes and the fine, white precipitate was collected first by centrifugation. The white solid was then washed with deionized water (2 x 50 mL) and centrifuged again. The collected precipitate was taken up in acetone (50 mL) and vacuum filtered through a fritted disc filter (medium porosity) and dried under vacuum for 30 minutes to furnish the methyl ester cyclodextrin intermediate as a pure off-white solid (1.6 g, 91%). Thus, methyl ester *β*-cyclodextrin intermediate (1.6 g, 0.86 mmol) was treated with 1 M KOH/H_2_O (6.45 mL, 6.45 mmol, 7.5 equiv. to cyclodextrin) in a 20 mL scintillation vial equipped with a stir bar. The initial suspension became a full solution (light tan in color) after 10 minutes of stirring at ambient temperature. The mixture was vigorously stirred overnight. The light tan solution was added dropwise to a stirring acetone bath (300 mL) in a 500 mL Erlenmeyer flask. White flakes precipitated out upon the addition of the mixture, and these were collected by centrifugation. Additionally, the white solid was re-suspended, washed with MeOH (2 x 50 mL) and collected by centrifugation. Lastly, the solid was vacuum filtered, washed with MeOH (2 x 20 mL) and dried under vacuum for 2 h. The procedure yielded pure heptakis(6-*S*-(2-carboxyethyl)-6-thio)cyclomaltoheptaose heptapotassium salt (SBX, **2**) (1.55 g, 89%). ^1^H NMR (D_2_O, 600 MHz) δ 5.05 (d, *J* = 3.0, 6H), 3.94 (td, *J* = 9.0, 1.8, 6H), 3.84 (t, *J* = 9.6, 6H), 3.56-3.53 (m, 12H), 3.09 (d, *J* = 12.0, 6H), 2.93 (dd, *J* = 13.8, 7.2, 6H), 2.80 (t, *J* = 7.2, 12H), 2.43 (td, *J* = 7.2, 3.6, 12H); ^13^C NMR (D_2_O, 150 MHz) δ 180.6, 101.4, 83.3, 73.0, 72.4, 72.4, 37.7, 33.4, 29.6; LC-MS(TOF): [M-2H]^2-^, *m/z =* 874.1705 (874.1635 calc.).

Heptakis(6-*S*-(3-carboxypropyl)-6-thio)cyclomaltoheptaose heptapotassium salt (SBX+1, **3**)

Heptakis-6-bromo-6-deoxy-*β*-cyclodextrin (1.0 g, 0.63 mmol) was made into a suspension in *N*-methyl-2-pyrrolidone (NMP, 20 mL) in a 100 mL round bottomed flask equipped with a stir bar. Upon gentle heating the cyclodextrin fully dissolved forming a light tan-colored solution. To this solution, cesium carbonate (2.1 g, 6.3 mmol, 10 equiv. to cyclodextrin) was added in small portions followed by the addition via syringe of methyl 4-sulfanylbutanoate (0.84 g, 6.3 mmol, 10 equiv. to cyclodextrin). The resulting mixture was heated to 55 ^o^C overnight with vigorous stirring. The following day, the suspension was cooled to ambient temperature and the mixture added dropwise to a vigorously stirring 500 mL Erlenmeyer flask filled with 300 mL of acetone to induce precipitation of the modified cyclodextrin. Stirring of the suspension was done at ambient temperature for 10 minutes and the fine, white precipitate was collected first by centrifugation. The white solid was then washed with deionized water (2 x 50 mL) and centrifuged again. The collected precipitate was taken up in acetone (50 mL) and vacuum filtered through a fritted disc filter (medium porosity) and dried under vacuum for 30 minutes to furnish the methyl ester *β*-cyclodextrin intermediate as a pure, off-white solid (1.07 g, 87%). Thus, methyl ester *β*-cyclodextrin intermediate (1.07 g, 0.55 mmol) was treated with 1 M KOH/H_2_O (4.13 mL, 4.13 mmol, 7.5 equiv. to cyclodextrin) in a 20 mL scintillation vial equipped with a stir bar. The initial suspension became a full solution (light tan in color) after 10 minutes of stirring at ambient temperature. The mixture was vigorously stirred overnight. The light tan solution was added dropwise to a stirring acetone bath (300 mL) in a 500 mL Erlenmeyer flask. White flakes precipitated out upon the addition of the mixture, and these were collected by centrifugation. Additionally, the white solid was re-suspended, washed with MeOH (2 x 50 mL) and collected by centrifugation. Lastly, the solid was vacuum filtered, washed with MeOH (2 x 20 mL) and dried under vacuum for 2 h. The procedure yielded pure heptakis(6-*S*-(3-carboxypropyl)-6-thio)cyclomaltoheptaose heptapotassium salt (SBX+1, **3**) (0.95 g, 82%). ^1^H NMR (D_2_O, 600 MHz) δ 5.12 (d, *J* = 3.0, 6H), 3.94 (t, *J* = 9.0, 6H), 3.89 (t, *J* = 9.6, 6H), 3.57-3.52 (m, 12H), 3.10 (d, *J* = 13.2, 6H), 2.92 (dd, *J* = 13.2, 7.8, 6H), 2.67-2.59 (m, 12H), 2.22 (t, *J* = 7.2, 12H), 2.16 (br s, 6H), 1.84-1.77 (m, 12H); ^13^C NMR (D_2_O, 150 MHz) δ 182.3, 100.0, 82.2, 72.8, 71.9, 71.3, 36.7, 33.4, 32.7, 26.2; LC-MS(TOF): [M-2H]^2-^, *m/z =* 923.2228 (923.2253 calc.).

Heptakis(6-*S*-(2-hydroxyethyl)-6-thio)cyclomaltoheptaose (SBN-1, **4**)

Heptakis-6-deoxy-6-bromo-*β*-cyclodextrin (1.2 g, 0.76 mmol) was made into a suspension with *N*-methyl-2-pyrrolidone (NMP, 20 mL) in a 100 mL round bottomed flask equipped with a stir bar. Upon gentle heating the cyclodextrin fully dissolved forming a light tan-colored solution. To this solution, cesium carbonate (2.48 g, 7.6 mmol, 10 equiv. to cyclodextrin) was added in small portions followed by the addition via syringe of 2-mercaptoethanol (0.53 mL, 0.59 g, 7.6 mmol, 10 equiv. to cyclodextrin). The resulting mixture was heated to 55 ^o^C overnight. The following day, the suspension was cooled to ambient temperature added dropwise to a vigorously stirring 500 mL Erlenmeyer flask filled with 300 mL of acetone to induce precipitation of the product. Stirring of the suspension was done at ambient temperature for 10 minutes and the fine precipitate (white flakes) were collected by centrifugation. The white precipitate was taken up in deionized water (50 mL) and the suspension was heated using a heat gun until the solid was fully solubilized. The solution was allowed to stand at ambient temperature for 2 hours, during which time a white, fine solid began to slowly precipitate. The suspension was centrifuged and the collected white solid (after carefully decanting the water layer) was washed with acetone (3 x 40 mL) and dried under vacuum for 4 hours. The process provides heptakis(6-*S*-(2-hydroxyethyl)-6-thio)cyclomaltoheptaose (SBN-1, **4**) in pure form as a flaky, white solid (0.97 g, 82%). ^1^H NMR (D_2_O, 600 MHz) δ 4.99 (d, *J* = 3.6, 6H), 3.80 (t, *J* = 9.6, 6H), 3.77 (t, *J* = 9.6, 6H), 3.62 (t, *J* = 6.0, 12H), 3.51 (dd, *J* = 10.2, 3.6, 6H), 3.41 (t, *J* = 9.6, 6H), 3.10 (d, *J* = 12.0, 6H), 2.43 (app dd, *J* = 13.8, 9.0, 6H), 2.72-2.68 (m, 12H); ^13^C NMR (D_2_O, 150 MHz) δ 100.9, 83.6, 72.7, 71.7, 71.2, 60.4, 34.6, 33.2; LC-MS(TOF): [M+FA-H]^-^, *m/z =* 1599.3931 (1599.3905 calc.)

Heptakis(6-*S*-(3-hydroxypropyl)-6-thio)cyclomaltoheptaose (SBN, **5**)

Heptakis-6-deoxy-6-bromo-*β*-cyclodextrin (1.0 g, 0.63 mmol) was made into a suspension with *N*-methyl-2-pyrrolidone (NMP, 20 mL) in a 100 mL round bottomed flask equipped with a stir bar. Upon gentle heating the cyclodextrin fully dissolved forming a light tan-colored solution. To this solution, cesium carbonate (2.1 g, 6.3 mmol, 10 equiv. to cyclodextrin) was added in small portions followed by the addition via syringe of 3-mercapto-1-propanol (0.54 mL, 0.58 g, 6.3 mmol, 10 equiv. to cyclodextrin). The resulting mixture was heated to 55 ^o^C overnight. The following day, the suspension was cooled to ambient temperature added dropwise to a vigorously stirring 500 mL Erlenmeyer flask filled with 300 mL of acetone to induce precipitation of the product. Stirring of the suspension was done at ambient temperature for 10 minutes and the fine precipitate (white flakes) were collected by centrifugation. The white precipitate was taken up in deionized water (50 mL) and the suspension was heated using a heat gun until the solid was fully solubilized. The solution was allowed to stand at ambient temperature for 2 hours, during which time a fine, white solid began to slowly precipitate. The suspension was centrifuged and the collected white solid (after carefully decanting the water layer) was washed with acetone (3 x 40 mL) and dried under vacuum for 4 hours. The process provides the neutral, heptakis(6-*S*-(3-hydroxypropyl)-6-thio)cyclomaltoheptaose (SBN, **5**) in pure form as a white solid (0.88 g, 84%). ^1^H NMR (D_2_O, 600 MHz) δ 5.07 (s, 6H), 3.88-3.83 (m, 12H), 3.63-3.60 (m, 18H), 3.48 (t, *J* = 9.0, 6H), 3.19 (d, *J* = 13.2, 6H), 2.72-2.68 (m, 12H), 1.80 (t, *J* = 5.4, 12H; ^13^C NMR (D_2_O, 150 MHz) δ 101.1, 84.0, 72.9, 71.8, 71.5, 60.4, 33.5, 31.7, 29.2; LC-MS(TOF): [M+FA-H]^-^, *m/z =* 1697.4975 (1697.5000 calc.)

Heptakis(6-*S*-(4-hydroxybutyl)-6-thio)cyclomaltoheptaose (SBN+1, **6**)

Heptakis-6-deoxy-6-bromo-*β*-cyclodextrin (1.1 g, 0.7 mmol) was made into a suspension with *N*-methyl-2-pyrrolidone (NMP, 20 mL) in a 100 mL round bottomed flask equipped with a stir bar. Upon gentle heating the cyclodextrin fully dissolved forming a light tan-colored solution. To this solution, cesium carbonate (2.3 g, 7.0 mmol, 10 equiv. to cyclodextrin) was added in small portions followed by the addition via syringe of methyl 4-mercapto-1-butanol (0.72 mL, 0.74 g, 7.0 mmol, 10 equiv. to cyclodextrin). The resulting mixture was heated to 55 ^o^C overnight. The following day, the suspension was cooled to ambient temperature added dropwise to a vigorously stirring 500 mL Erlenmeyer flask filled with 300 mL of acetone to induce precipitation of the product. Stirring of the suspension was done at ambient temperature for 10 minutes and the fine precipitate (white flakes) were collected by centrifugation. The white precipitate was taken up in deionized water (50 mL) and the suspension was heated using a heat gun until the solid was fully solubilized. The solution was allowed to stand at ambient temperature for 2 hours, during which time a fine, white solid began to slowly precipitate. The suspension was centrifuged and the collected white solid (after carefully decanting the water layer) was washed with acetone (3 x 40 mL) and dried under vacuum for 4 hours. The process provides heptakis(6-*S*-(4-hydroxybutyl)-6-thio)cyclomaltoheptaose (SBN+1, **6**) in pure form as a white solid (1.04 g, 85%). ^1^H NMR (D_2_O, 600 MHz) δ 5.05 (d, *J* = 3.6, 6H), 3.86-3.81 (m, 12H), 3.61 (dd, *J* = 9.6, 3.6, 6H), 3.55 (t, *J* = 6.0, 12H), 3.46 (t, *J* = 9.0, 6H), 3.20 (d, *J* = 12.6, 6H), 2.86 (dd, *J* = 13.8, 9.6, 6H), 2.72-2.64 (m, 12H), 1.64-1.59 (m, 24H); ^13^C NMR (D_2_O, 150 MHz) δ 101.2, 84.2, 73.0, 71.9, 71.7, 61.3, 33.5, 32.7, 31.0, 25.8; LC-MS(TOF): [M+FA-H]^-^, *m/z =* 1795.6126 (1795.6069 calc.).

Hexakis(6-*S*-(2-carboxyethyl)-6-thio)cyclomaltohexaose hexapotassium salt (SAX, **7**)

Hexakis-6-bromo-6-deoxy-*α*-cyclodextrin (1.6 g, 1.18 mmol) was made into a suspension in *N*-methyl-2-pyrrolidone (NMP, 20 mL) in a 100 mL round bottomed flask equipped with a stir bar. Upon gentle heating the cyclodextrin fully dissolved forming a light tan-colored solution. To this solution, cesium carbonate (3.84 g, 11.8 mmol, 10 equiv. to cyclodextrin) was added in small portions followed by the addition via syringe of methyl 3-mercaptopropionate (1.3 mL, 1.42 g, 11.8 mmol, 10 equiv. to cyclodextrin). The resulting mixture was heated to 55 ^o^C overnight with vigorous stirring. The following day, the suspension was cooled to ambient temperature and the mixture added dropwise to a vigorously stirring 500 mL Erlenmeyer flask filled with 300 mL of acetone to induce precipitation of the modified cyclodextrin. Stirring of the suspension was done at ambient temperature for 10 minutes and the fine, white precipitate was collected first by centrifugation. The white solid was then washed with deionized water (2 x 50 mL) and centrifuged again. It was found that two washings followed by centrifugation were sufficient to remove all the residual cesium carbonate and NMP. The collected precipitate was taken up in acetone (50 mL) and vacuum filtered through a fritted disc filter (medium porosity) and dried under vacuum for 30 minutes to furnish the methyl ester cyclodextrin intermediate as a pure off-white solid (1.65 g, 88%).^[[1]](#endnote-1)^ Methyl ester α-cyclodextrin intermediate (1.65 g, 1.04 mmol) was treated with 1 M KOH/H_2_O (7.8 mL, 7.8 mmol, 7.5 equiv. to cyclodextrin) in a 20 mL scintillation vial equipped with a stir bar. The initial suspension became a full solution (light tan in color) after 10 minutes of stirring at ambient temperature. The mixture was vigorously stirred overnight. The light tan solution was added dropwise to a stirring acetone bath (300 mL) in a 500 mL Erlenmeyer flask. White flakes precipitated out upon the addition of the mixture, and these were collected by centrifugation. Additionally, the white solid was re-suspended, washed with MeOH (2 x 50 mL) and collected by centrifugation. The additional methanol wash was found to efficiently remove any remaining KOH. Lastly, the solid was vacuum filtered, washed with MeOH (2 x 20 mL) and dried under vacuum for 2 h. The procedure yielded pure hexakis(6-*S*-(2-carboxyethyl)-6-thio)cyclomaltohexaose hexapotassium salt (SAX, **7**) (1.49 g, 83%). ^1^H NMR (D_2_O, 600 MHz) δ 5.04 (s, 6H), 3.99 (br s, 6H), 3.89 (t, *J* = 7.8, 6H), 3.57-3.53 (m, 12H), 3.11 (d, *J* = 13.2, 6H), 2.92 (dd, *J* = 13.2, 6.6, 6H), 2.80 (t, *J* = 7.8, 12H), 2.43 (td, *J* = 7.8, 12H); ^13^C NMR (D_2_O, 150 MHz) δ 180.6, 100.7 (br), 83.1 (br), 73.2, 71.9, 71.3, 37.7, 33.5, 29.4; LC-MS(TOF): [M-2H]^2-^, *m/z =* 749.1440 (749.1450 calc.).

Octakis(6-*S*-(2-carboxyethyl)-6-thio)cyclomaltooctaose octapotassium salt (SGX, **8**)

Octakis-6-bromo-6-deoxy-*γ*-cyclodextrin (1.6 g, 0.89 mmol) was made into a suspension in *N*-methyl-2-pyrrolidone (NMP, 20 mL) in a 100 mL round bottomed flask equipped with a stir bar. Upon gentle heating the cyclodextrin fully dissolved forming a light tan-colored solution. To this solution, cesium carbonate (2.9 g, 8.9 mmol, 10 equiv. to cyclodextrin) was added in small portions followed by the addition via syringe of methyl 3-mercaptopropionate (0.99 mL, 1.07 g, 8.9 mmol, 10 equiv. to cyclodextrin). The resulting mixture was heated to 55 ^o^C overnight with vigorous stirring. The following day, the suspension was cooled to ambient temperature and the mixture added dropwise to a vigorously stirring 500 mL Erlenmeyer flask filled with 300 mL of acetone to induce precipitation of the modified cyclodextrin. Stirring of the suspension was done at ambient temperature for 10 minutes and the fine, white precipitate was collected first by centrifugation. The white solid was then washed with deionized water (2 x 50 mL) and centrifuged again. The collected precipitate was taken up in acetone (50 mL) and vacuum filtered through a fritted disc filter (medium porosity) and dried under vacuum for 30 minutes to furnish the methyl ester *γ*-cyclodextrin intermediate as a pure off-white solid (1.75 g, 93%). Thus, methyl ester *γ*-cyclodextrin intermediate (1.75 g, 0.83 mmol) was treated with 1 M KOH/H_2_O (6.3 mL, 6.3 mmol, 7.5 equiv. to cyclodextrin) in a 20 mL scintillation vial equipped with a stir bar. The initial suspension became a full solution (light tan in color) after 10 minutes of stirring at ambient temperature. The mixture was vigorously stirred overnight. The light tan solution was added dropwise to a stirring acetone bath (300 mL) in a 500 mL Erlenmeyer flask. White flakes precipitated out upon the addition of the mixture and these were collected by centrifugation. Additionally, the white solid was washed with MeOH (2 x 50 mL) and collected by centrifugation. Lastly, the solid was vacuum filtered, washed with MeOH (2 x 20 mL) and dried under vacuum for 2 h. The procedure yielded pure octakis(6-*S*-(2-carboxyethyl)-6-thio)cyclomaltooctaose octapotassium salt (SGX, **8**) (1.65 g, 86%). ^1^H NMR (D_2_O, 600 MHz) δ 5.12 (s, 6H), 4.00 (br s, 6H), 3.89 (d, *J* = 9.2, 6H), 3.60-3.57 (m, 12H), 3.07 (d, *J* = 14.2, 12H), 2.95 (dd, *J* = 14.2, 5.9, 6H), 2.80 (t, *J* = 7.1, 12H), 2.45-2.42 (m, 12H); ^13^C NMR (D_2_O, 150 MHz) δ 180.6, 101.0, 82.3, 72.6, 72.3, 71.2, 37.7, 33.4, 29.6; LC-MS(TOF): [M-2H]^2-^, *m/z =* 999.1925 (999.1961 calc.)

**Formalism for “Self-Competitive” (Two-state) Binding Model**

Establishing an analytical formalism for a two-state binding system begins with expressions for the equilibrium binding constants and host/guest mass balances.^[[2]](#endnote-2)^

$$K_{u}=\frac{[HG_{u}]}{\left[ H \right][G_{u}]} K_{d}=\frac{[HG_{d}]}{\left[ H \right][G_{d}]}$$

$$\left[ H \right]_{0}=\left[ H \right]+\left[ HG_{u} \right]+\left[ HG_{d} \right]$$

$${[G_{u}]}_{0}=\left[ G_{u} \right]+\left[ HG_{u} \right] {[G_{d}]}_{0}=\left[ G_{d} \right]+\left[ HG_{d} \right]$$

$$\left[ G \right]_{0}=\left[ G_{u} \right]_{0}+\left[ G_{d} \right]_{0}$$

$$\left[ G_{u} \right]_{0}=\left[ G_{d} \right]_{0}$$

Two expressions for *K* are required, one for each fentanyl configuration: *u* = “up” and *d* = “down.” [*H*] is the concentration of free host. [*G_u_*] and [*G_d_*] are the concentrations of free guest in the up and down configurations, respectively. [*HG_u_*] and [*HG_d_*] are the concentrations of the up and down fentanyl:CD complexes, respectively. Though there is little physical meaningfulness of these concentrations, they must be invoked for the completeness of the model. The subscript “0” refers to the initial total concentration of a particular species. The last equality simply reflects that the up and down states are equally likely due to an isotropic motion of fentanyl in solution. Using the equations, one can obtain a cubic polynomial in terms of *free host* [*H*], the roots of which must be found.

**Solving for Cubic Roots**

This method for solving for roots of cubic equations is based on work and information presented previously.^[[3]](#endnote-3)^^[[4]](#endnote-4)^ Note that for the Tschirnhaus-Vieta approach, all three cubic roots are real if *f* < 0 (as given below). Physically relevant/realizable values for *K*_11_ and *K*_21_ are only possible for this numerical fitting algorithm if this condition is satisfied for the entire range of experimental [*G*]_0_ and [*H*]_0_ values. It is important to emphasize that the treatment below is strictly for 1:1 binding equilibria where the compound that binds can adopt in one of two orientations (that cannot be distinguished between when free in solution).

In physical variable space:

$$\boldsymbol{a}\left[ \boldsymbol{H} \right]^{\boldsymbol{3}}\boldsymbol{+b}\boldsymbol{[H]}^{\boldsymbol{2}}\boldsymbol{+c}\left[ \boldsymbol{H} \right]\boldsymbol{+d=0}$$

$$\boldsymbol{a= K}_{\boldsymbol{u}}\boldsymbol{K}_{\boldsymbol{d}}$$

$$\boldsymbol{b=}\left( \boldsymbol{K}_{\boldsymbol{u}}\boldsymbol{+}\boldsymbol{K}_{\boldsymbol{d}} \right)\boldsymbol{+}\boldsymbol{K}_{\boldsymbol{u}}\boldsymbol{K}_{\boldsymbol{d}}\boldsymbol{(}\left[ \boldsymbol{G} \right]_{\boldsymbol{0}}\boldsymbol{-}\boldsymbol{[H]}_{\boldsymbol{0}}\boldsymbol{)}$$

$$\boldsymbol{c=1+(}\boldsymbol{K}_{\boldsymbol{u}}\boldsymbol{+}\boldsymbol{K}_{\boldsymbol{d}}\boldsymbol{)}\left( \left[ \boldsymbol{G} \right]_{\boldsymbol{0}}\boldsymbol{-}\left[ \boldsymbol{H} \right]_{\boldsymbol{0}} \right)$$

$$\boldsymbol{d=-}\left[ \boldsymbol{H} \right]_{\boldsymbol{0}}$$

Variable Transformation:

$$f=\frac{1}{3}\left( \frac{3c}{a}-\frac{b^{2}}{a^{2}} \right)$$

$$g=\frac{1}{27}\left( \frac{2b^{3}}{a^{3}}-\frac{9bc}{a^{2}}+\frac{27d}{a} \right)$$

$$h=\frac{g^{2}}{4}+\frac{f^{3}}{27}$$

$$i=\sqrt{\frac{g^{2}}{4}-h}$$

$$j=\sqrt[3]{i}$$

$$k=\arccos\left( \frac{-g}{2i} \right)$$

$$L=-j$$

$$M=\cos\left( \frac{k}{3} \right)$$

$$N=\sqrt{3}\sin\left( \frac{k}{3} \right)$$

$$P=\frac{-b}{3a}$$

Arriving at final cubic roots:

$$\chi_{1}=2j\cos\left( \frac{k}{3} \right)+P$$

$$\chi_{2}=L\left( M+N \right)+P$$

$$\chi_{3}=L\left( M-N \right)+P$$

Physically relevant solution is the maximum root:

$$\left[ \boldsymbol{H} \right]\boldsymbol{=}\max_{\boldsymbol{i}} \boldsymbol{\chi}_{\boldsymbol{i}}$$

**Parameter Correlation and Impact on Robustness of Fit**

Correlation matrices are a useful indicator of the interrelationships between variables. For the current work these matrices show that the extraction of *K*_1_ was robust for all fittings were the competitive 1:1 model was required/used to fit the NMR titration data. Other parameters were much more sensitive to variations in the data *and* to correlations between parameters. This is due, in part to the large disparities between *K*_1_ and *K*_2_ (generally an order of magnitude). This difference results in large population differences in the “up” and “down” conformations. These differences, however, may have large effects on local physical and electronic structure of the complex. These effects may bring to bear significant changes in chemical shift even though *K*_2_ may be small, which result in observable non-monotonic behavior, particularly for Job plot data.

**NMR Job Plots for Non-1:1 Complexation**

Given below are Job plots for the two host cyclodextrins that displayed non-1:1 binding modalities: SBX and SBX+1. Note that several of the curve maxima are *not* at 0.5, the value expected for 1:1 complexes. This behavior is more pronounced for the SBX+1 data. Also, though a subtler feature, note the inflection point in the curvature for the aromatic data (triangles). This behavior is only observed for binding modalities more complex than simple 1:1 systems.

**Supplementary Figure 1.** Job plots for SBX (bottom) and SBX+1 (top) complexes with fentanyl. Diamonds: H5 protons, Squares: H2 protons, Triangles: Amide-end aromatic protons.

**Example ^1^H NMR Titration Data**

Below shows representative NMR titration data displaying clear behavior that binding is more complicated than a simple 1:1 titration (see nonmonotonic behavior of 3.8908 and 3.8481 ppm peaks). Fitting such data could only be done assuming a two-state 1:1 binding mode. This mode is reflective of a guest’s binding to one of two sites on a host *or* – as in the case here – the guest’s being able to adopt one of two confirmations within the CD interior.

**Supplementary Figure 2.** Titration plot for SBX+1 and fentanyl. Chemical shift data have been referenced to the chemical shift at 0 mM fentanyl.

**NMR ROESY Data**

**Supplementary Figure 3.** Full Two-dimensional ROESY spectrum of a 1:3.7 fentanyl HCl + SBX+1 mixture.

**Supplementary Figure 4.** Two-dimensional ROESY spectrum of a 1:3.7 fentanyl HCl + SBX mixture.

1. In the case of the SBX and other carboxylic acid analogs synthesized, the intermediate methyl esters were obtained in >98% purity (by ^1^H NMR in DMSO-*_d6_*), and thus were just carried onto the next step (hydrolysis). We provide the NMR data for all final compounds in D_2_O. [↑](#endnote-ref-1)
2. Thordarson, P. *Chem. Soc. Rev.* **2011**, *40*, 1305-1323. [↑](#endnote-ref-2)
3. Abramowitz, A. Elementary Analytical Methods. In *Handbook of Mathematical Functions: with Formulas, Graphs, and Mathematical Tables;* Abramowitz, M., Stegun, I. A.; Dover Publications, Inc.: New York, NY, 1965. [↑](#endnote-ref-3)
4. Cubic Formula. <http://mathworld.wolfram.com/CubicFormula.html> (accessed May 2016). [↑](#endnote-ref-4)
